# Anabolic Steroid-Induced Cholestatic Liver Injury: A Case Report

**DOI:** 10.7759/cureus.27514

**Published:** 2022-07-31

**Authors:** Osama Qasim Agha, Hussam Al Hennawi, Mustafa Alani, Shehroz Aslam, Justin Reynolds

**Affiliations:** 1 Internal Medicine, Creighton University School of Medicine, Phoenix, USA; 2 Internal Medicine, St. Joseph's Hospital and Medical Center, Phoenix, USA; 3 Internal Medicine, University of Arizona College of Medicine, Phoenix, USA; 4 Internal Medicine, Jefferson Abington Hospital, Abington, USA; 5 Gastroenterology, Creighton University School of Medicine, Phoenix, USA; 6 Hepatology, St. Joseph's Hospital and Medical Center, Phoenix, USA

**Keywords:** drug-induced liver injury, nandrolone, exogenous testosterone use, cholestatic liver injury, anabolic-androgenic steroids, anabolic steroids

## Abstract

Owing to performance-enhancing and cosmetic effects, illicit use of anabolic-androgenic steroids (AAS) has been well-described and can be associated with significant complications. We report a 27-year-old Caucasian male who self-medicated with AAS in the form of intramuscular injections and oral testosterone for a one-year duration. He complained of persistent jaundice and moderate generalized itching for one month. On admission, his total bilirubin level was 11.4 mg/dl (normal: 0-1.2 mg/dl), and liver enzymes were slightly elevated. On follow-up, the patient stated complete resolution of symptoms and near-normalization of lab results after one month of conservative management.

## Introduction

Anabolic-androgenic steroids (AAS) are synthetic compounds that resemble the male hormone testosterone and have an anabolic and masculinizing effect. Expert bodybuilders and young amateur athletes sometimes use AAS to increase muscle size and strength [1]. The use of such compounds started in the 1960s primarily for aesthetic purposes owing to the desired performance-enhancing effect [2]. At present, AAS nonmedical use has expanded globally, mainly among males with no athletic background who find it a shortcut for physical appearance and strength goals [3]. In fact, AAS athletic use currently constitutes the minority of consumers for recreational and aesthetic purposes. An epidemiologic meta-analysis demonstrated a 3.3% global lifetime prevalence rate with a 6.4% prevalence rate among males compared with 1.6% among females [4].

Short-term use of AAS may associate with a few considerable side effects compared to long-term consumption that may interfere with hormonal homeostasis resulting in physical and psychological manifestations as well as increased mortality [5-7]. Some of these manifestations include oligospermia, testicular atrophy, male pattern baldness, left ventricle hypertrophy, liver tumors, and liver damage [5-7]. Similar to other disease entities, patients with toxic or drug-induced liver injury may present with jaundice due to significant elevation of bilirubin levels, albeit near-normal liver enzyme levels. Here, we report a case of drug-induced liver injury (DILI) due to the illicit use of AAS.

This case was presented as a poster at the American College of Gastroenterology meeting in October 2021.

## Case presentation

**Figure 1:**
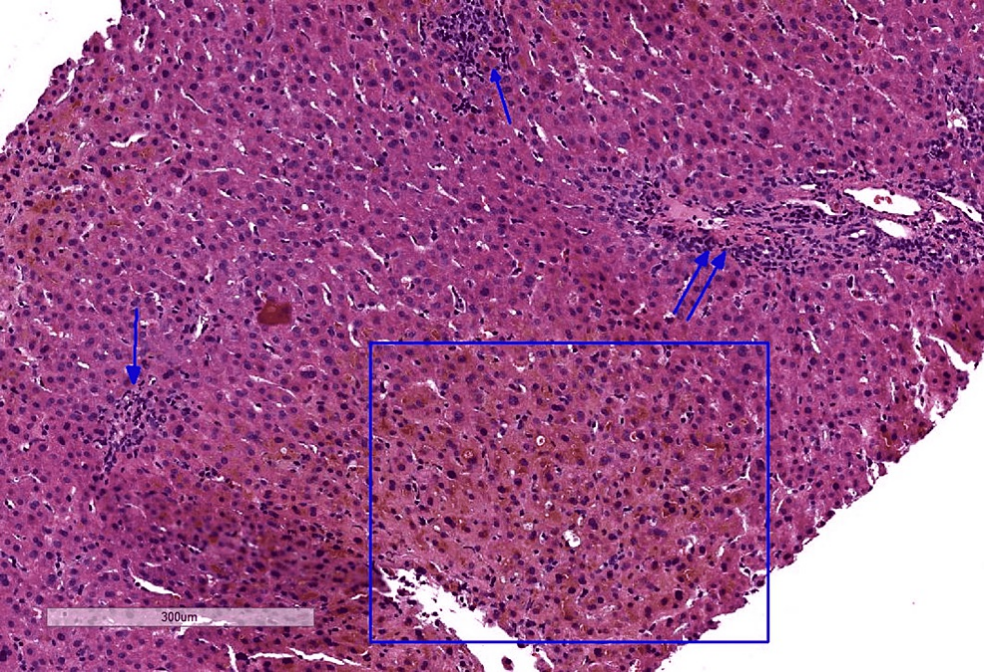
A liver biopsy showed cholestatic hepatitis with mild inflammatory activity. Single arrows indicate foci of lobular inflammation, and double arrows indicate a minimally inflamed portal tract. The square indicates an area with centrilobular/zone 3 cholestasis with hepatocellular bile accumulation and canalicular bile plugs.

A 27-year-old Caucasian male presented to our facility with persistent jaundice and generalized itching for a one-month duration. He presented to another hospital when his symptoms started one month earlier, and he was advised conservative management. Due to the persistence of his symptoms and lack of improvement, he later presented to our facility for a second opinion. He denied chronic medical problems or taking any prescribed medications. He had been injecting anabolic-androgenic steroids (Nandrolone) intramuscularly once weekly for one year until the onset of his symptoms and consuming oral testosterone to enhance muscle mass. He denied any alcohol abuse or IV drug use. Physical examination was remarkable for scleral icterus and jaundice. There were no stigmata of chronic liver disease or portal hypertension. His labs were significant for total bilirubin 11.4 mg/dl (normal: 0-1.2 mg/dl), direct bilirubin 8.4 mg/dl (normal: 0-0.5 mg/dl), alanine transaminase (ALT) 52 U/L (normal: 6-44 U/L), aspartate aminotransferase (AST) 47 U/L (normal: 5-34 U/L), and alkaline phosphatase (ALP) 190 U/L (normal: 40-150 U/L). Complete blood count (CBC), international normalized ratio (INR), acute viral hepatitis panel, antinuclear antibodies (ANA), antimitochondrial antibodies (AMA), anti-actin antibodies, and ceruloplasmin levels were all within normal limits. Abdominal ultrasound and magnetic resonance imaging (MRI) were remarkable for a likely benign hemangioma with no cholelithiasis or other acute abnormalities. Concurrently, DILI secondary to AAS intake was suspected with an R score of 0.82 indicative of cholestatic injury. Due to the persistence of jaundice for one month despite stopping AAS, a percutaneous liver biopsy was performed and showed cholestatic hepatitis with mild inflammatory activity (Figure 1). Using the Council for International Organizations of Medical Scientists/Roussel Uclaf Assessment Method (CIOMS/RUCAM) scale, a score of 3 was calculated as indicative of "possible" causality [8].

**Figure 2:**
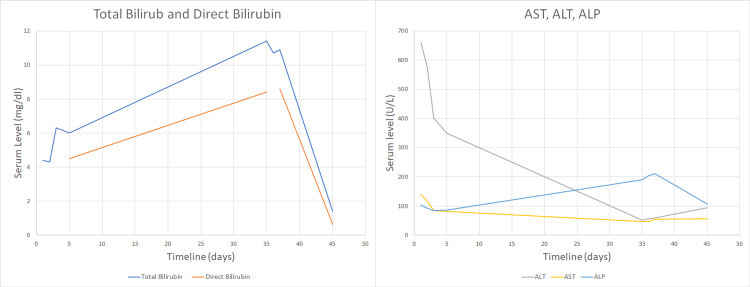
Trend in the liver function tests ALT: Alanine transaminase; AST: Aspartate aminotransferase; ALP: Alkaline phosphatase.

A diagnosis of AAS DILI was made, and the patient was counseled about the importance of cessation of all anabolic steroids. Ursodeoxycholic acid (UDCA) and hydroxyzine were prescribed for symptom control. One month later, marked improvement in his labs and near-normalization of his bilirubin levels were evident (Figure 2 and Table 1).

**Table 1:** Liver function tests one month prior to presentation, at the time of presentation, and outpatient follow-up T Bili: Total bilirubin; D Bili: Direct bilirubin; ALT: Alanine transaminase; AST: Aspartate aminotransferase; ALP: Alkaline phosphatase; N/A: Not available.

Lab	1 month prior	At presentation	1-month follow-up	Normal range
T Bili	4.4	11.4	1.4	0-1.2 mg/dl
D Bili	N/A	8.4	0.64	0-0.5 mg/dl
ALT	663	52	94	6-44 U/L
AST	140	47	57	5-34 U/L
ALP	103	190	106	40-150 U/L

## Discussion

Synthetic anabolic steroids are readily available over-the-counter products and are easily accessible through online stores, with consumption rates increasing over the year [8]. Several formulations and routes of administration of AAS have been discussed in the literature with a specific complication that may follow the intake of particular compounds over others [8].

Androgens and other steroidal compounds exhibit their effects via binding to intracellular receptors and collective translocation to nuclear targets. Binding to androgen response elements on DNA initiates a cascade of events resulting in cellular growth and differentiation. Stemming from such effect, hepatocytes undergo a series of unregulated growth and differentiation giving rise to hepatic tumors and nodular regeneration following anabolic steroid consumption. On the other hand, cholestatic changes associated with the C-17α alkylated form of androgens intake are not well understood but are observed similarly in animal models upon consumption of high dosages [9]. Similarly, this phenomenon can occur in patients with cholestatic changes associated with pregnancy or oral contraceptive pills and estrogen intake with the postulated rationale being changes in or lack of the bile salt carrier proteins [10].

Four distinctive forms of hepatic injury can be associated with AAS. These include (1) a transient increase in serum liver enzymes, (2) acute cholestatic injury, (3) chronic vascular injury, and (4) hepatic tumors including hepatocellular carcinomas and benign adenomas. Notably, most of these side effects have been linked with the C-17α alkylated form of testosterone. Patients with AAS-induced acute cholestatic liver injury usually present with jaundice, pruritus, nausea, and fatigue. The extent of liver injury ranges from minor, transient enzymatic elevations to severe and long-term cholestasis. Distinct liver parenchymal injury may also occur with significantly elevated liver enzymes. In cholestatic liver injury, bilirubin is usually significantly elevated. Consistent with our case, “bland cholestasis” tends to be associated with minimal or mild inflammation usually seen on liver biopsy. The combination of UDCA and S-adenosylmethionine (SAMe) in the cholestatic liver may alleviate pruritis and help normalize bilirubin levels over the treatment course compared to placebo [3].

Our case clinical presentation, physical examination, and supplementary workup coupled with recovery upon conservative management correspond to mild cholestasis secondary to anabolic steroids intake, Nandrolone in our case. Of note, our patient had a minimal elevation of liver enzymes, but bilirubin was greatly elevated, reflective of bland cholestasis with minimal hepatocellular injury. The long-term prognosis of DILI attributed to AAS depends upon the initial clinical presentation and laboratory findings [5]. In our case and other milder forms of DILI, the prognosis is overall excellent with the cessation of the AAS although recovery might be prolonged.

## Conclusions

The illicit use of AAS in athletic males and bodybuilders can lead to various side effects including four different forms of liver injury. One form is characterized by bland cholestasis accompanied by minimal elevation of liver enzymes. Presenting symptoms of AAS-induced cholestatic liver injury are usually jaundice, pruritus, nausea, and fatigue. Early diagnosis of this condition is crucial given that conservative management with early cessation of AAS can lead to recovery, although it can be prolonged.
